# A Monte Carlo evaluation of beam characteristics for total body irradiation at extended treatment distances

**DOI:** 10.1120/jacmp.v15i3.4708

**Published:** 2014-05-08

**Authors:** Roumiana Chakarova, Marcus Krantz

**Affiliations:** ^1^ Department of Medical Physics and Biomedical Engineering Sahlgrenska University Hospital, Gothenburg, and Department of Radiation Physics, Sahlgrenska Academy, University of Gothenburg Sweden

**Keywords:** extended SSD, TBI, Monte Carlo simulations

## Abstract

The aim is to study beam characteristics at large distances when focusing on the electron component. In particular, to investigate the utility of spoilers with various thicknesses as an electron source, as well as the effect of different spoiler‐to‐surface distances (STSD) on the beam characteristics and, consequently, on the dose in the superficial region. A MC model of a 15 MV Varian accelerator, validated earlier by experimental data at isocenter and extended distances used in large‐field total body irradiation, is applied to evaluate beam characteristics at distances larger than 400 cm. Calculations are carried out using BEAMnrc/DOSXYZnrc code packages and phase space data are analyzed by the beam data processor BEAMdp. The electron component of the beam is analyzed at isocenter and extended distances, with and without spoilers as beam modifiers, assuming vacuum or air surrounding the accelerator head. Spoiler thickness of 1.6 cm is found to be optimal compared to thicknesses of 0.8 cm and 2.4 cm. The STSD variations should be taken into account when treating patients, in particular when the treatment protocols are based on a fixed distance to the patient central sagittal plane, and also, in order to maintain high dose in the superficial region.

PACS numbers: 87.55.D‐, 87.55.de, 87.55.K‐

## I. INTRODUCTION

High‐dose total body irradiation (TBI) is administered as a pretreatment modality for bone marrow transplantation for hematological malignancies and other disorders. There is no consensus in existing clinical protocols about total dose, dose rate, and fractionation schemes. Dose delivery is not harmonized, either. Traditionally, a large‐field technique at extended source‐to‐skin distances (SSD) of 400‐500 cm is implemented with lateral and/or anterior‐posterior high‐energy photon beams.^1^, ^2^, ^3^ Compensators are used to counteract the variations in patient thickness and tissue heterogeneities, and to improve the dose uniformity throughout the whole body. Plastic barriers (spoilers) are introduced in the beam generating secondary electrons to improve the dose coverage in the superficial region.^4^, ^5^

Alternative treatments at shorter (120‐190 cm) SSD involve a translating couch technique, also with beam aperture modulation on a more advanced level.^6^, ^7^, ^8^, ^9^ Modulated‐arc TBI has been recently applied^(10)^ utilizing an arc of static open‐field beams to irradiate patients as they lay on a stationary couch beneath the gantry, with blocks to provide shielding of organs at risk. In general, improved dose uniformity can be achieved with novel techniques. However, it requires dynamic interface between the couch movement and the accelerator beam delivery, and commissioning of the treatment planning system at distances of approximately 200 cm with corresponding validation of the dose calculation algorithms.

The traditional large‐field technique is still attractive due to its simplicity and applicability to most standard accelerators and also because of a relative larger clinical experience with TBI at low dose rate. As long as this technique is used in the clinics, it is appropriate to continue improve its elements. For example, a Monte Carlo (MC) method can be applied to obtain 3D dose distribution in the patient when a dedicated TBI option is not available in the clinical treatment planning system.^(11)^ Other aspects are related to the utilization of spoilers. The effect of 1 cm thick spoiler on the surface dose for 15 MV beams at extended treatment distances is investigated in Kassaee et al.^(5)^ However, regarding an optimal spoiler thickness, no conclusion is made. The beam characteristics concerning various spoilers as a replacement for surface and lateral bolus are studied for 18 MV beams at 200 cm distances in Gerig et al.^(9)^ Also, the effect of spoiler‐to‐surface distances (STSD) on the buildup region is considered in the work cited. Complementary studies are needed, addressing optimization of the spoiler thickness, as well as the influence of various STSD on the buildup region for a 15 MV photon beam at SSD larger than 400 cm.

A bilateral beam delivery is implemented in our clinic, with patients lying on a couch behind a spoiler at 480 cm distance between the linear accelerator target and the patient central sagittal plane. A single 15 MV rectangular field, defined by a multileaf collimator and block, is used to treat all patients. The implementation of a MC method to simulate the treatment and to investigate 3D dose distributions in patients has been reported earlier.^(11)^ This work applies the same MC method for analysis of beam characteristics at large distances, focusing on the electron component. Thus, the aim is to investigate the utility of various spoiler thicknesses as an electron source, as well as the effect of different STSD on the beam characteristics and, consequently, the dose in the superficial region.

A MC model of a 15 MV Varian Clinac iX linear accelerator (Varian Medical Systems Inc., Palo Alto, CA) has been used to study beam characteristics at extended distances for large‐fields TBI technique. The electron component of the beam is analyzed at short (90 cm) and extended (445 cm) distances, with and without spoilers as beam modifiers.

**Figure 1:**
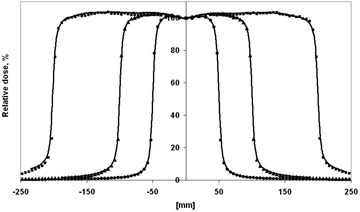
Cross profiles in water for $10\times 10\;{\text{cm}}^{2}$, $20\times 20\;{\text{cm}}^{2}$, and $40\times 40\;{\text{cm}}^{2}$ fields at SSD=90 cm. Lines: measured data; symbols: MC data.

Calculations are carried out using BEAMnrc/DOSXYZnrc code packages^12^, ^13^ for a 15 MV Varian Clinac iX linear accelerator. The model is based on the technical data provided. Energy and spatial distribution of the electrons incident on the target are determined by comparison of calculated data and measurements in water at SSD=90 cm performed at accelerator commissioning. Depth dose distributions and profiles at different depths are considered for field sizes up to $40\times 40\;{\text{cm}}^{2}$. The optimum parameter set established is the following: a nominal energy of the incident electrons of 14.4 MeV with 3% energy spread and a Gaussian spatial distribution with 0.1 cm focal spot FWHM. Validation profiles for $10\times 10\;{\text{cm}}^{2}$, $20\times 20\;{\text{cm}}^{2}$, and $40\times 40\;{\text{cm}}^{2}$ fields are shown in Fig. 1.

The capability of the accelerator model for extended SSD is verified by ion chamber measurements reported elsewhere.(11) In the work cited, calculated and measured profile and depth dose distributions are compared in a large water phantom at SSD=460 cm as well as in a CIRS thorax phantom (CIRS Tissue Simulation Technology, Norfolk, VA) equipped by water, lung, and bone‐equivalent inserts with ion chamber cavity.

Here, the beam characteristics are investigated by producing phase space files at 90 cm and at 445 cm distance for a $13\times 37\;{\text{cm}}^{2}$ rectangular field, with field size defined at isocenter distance. A 445 cm distance is determined by the position of the spoiler holder in our clinic. Jaws are opened to $39\times 39\;{\text{cm}}^{2}$ field size. The rectangular field is shaped by a block at the MLC location. This geometry is considered to reproduce head scattering conditions representative for the clinical treatment. Phase space files are calculated assuming air or vacuum surrounding the accelerator. The files are further analyzed by the beam data processor, BEAMdp.^(14)^ The areas used in the analysis are $34\times 34\;{\text{cm}}^{2}$ and $168\times 168\;{\text{cm}}^{2}$ for phase space at 90 cm and at 445 cm or larger distance, respectively. Energy fluence distributions and angular distributions are calculated, including all electrons registered in the phase space within the corresponding area without spatial differentiation. Spatial information is taken into account when deriving the mean electron energy as a function of the radial (off‐axis) distance.

**Figure 2:**
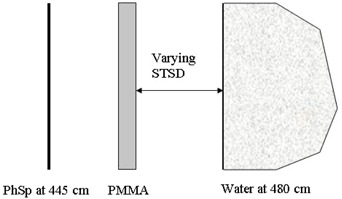
MC geometry for calculation of central depth dose profile for different spoiler‐to‐surface distances (STSD). Phase space is produced at 445 cm from the accelerator target. Source‐to‐water surface distance is fixed to 480 cm; STSD varies.

Effects of the extended irradiation distance and the presence of a spoiler are investigated by comparison of the beam characteristics at isocenter and extended distances, with and without a spoiler. PMMA spoilers with different thicknesses (0.8 cm, 1.6 cm and 2.4 cm) are available in our clinic and these spoilers are simulated with a surface towards the irradiated geometry at 445 cm. Subsequently, the phase space files produced at this distance are further used for depth dose calculations in water. The dose calculations are performed by the DOSXYZnrc routine with a voxel size of $2.0\times 2.0\times 0.5\;{\text{cm}}^{3}$ along the central axis of the beam. A fixed distance (SSD=480 cm) to the water surface is assumed when varying STSD to accommodate STSD up to 30 cm (Fig. 2).

Calculations are carried out on a Linux cluster at the National Supercomputer Centre Linköping, Sweden. The statistical uncertainty (one standard deviation) is indicated in the figures presenting the data.

## III. RESULTS & DISCUSSION

**Figure 3:**
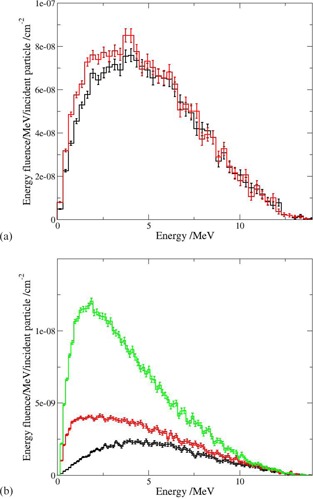
Characteristics of the electron component of the beam (estimated real fluence) at (a) SSD=90 cm and (b) SSD=445 cm. Black histograms: vacuum surrounding the accelerator head; red histograms : air surrounding the accelerator head; green histogram: air surrounding the accelerator head and presence of 1.6 cm PMMA spoiler.

**Figure 4:**
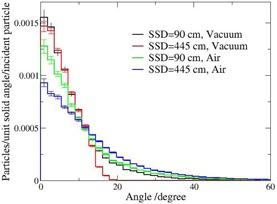
Angular distribution of the electron component of the beam. Black histogram: vacuum surrounding the accelerator head, SSD=90 cm; red histogram: vacuum surrounding the accelerator head, SSD=445 cm; green histogram: air surrounding the accelerator head, SSD=90 cm; blue histogram: air surrounding accelerator head, SSD=445 cm.

Energy fluence and angular distribution of electrons at short (90 cm) and extended (445 cm) distances are presented in Fig. 3 and Fig. 4. Vacuum or air is assumed surrounding the accelerator. There are mainly head scattered electrons at the short distance. The contribution of electrons generated in the air is small and the energy fluence is similar to that for vacuum with a maximum energy of approximately 4 MeV (Fig. 3(a)).

More electrons are produced in the thick air layer and the electron fluence in that case (Fig. 3(b), the red histogram) is much higher compared to that for vacuum. As shown in Petti et al.^(15)^ for 25 MV beams, a significant part of the electrons from the treatment head are passing through the air and are reaching 400 cm. These contamination electrons are produced in high‐Z components, such as flattening filter and jaws. Their energy is reduced when penetrating the air. Simultaneously, the number of electrons generated along the irradiated air path, mostly by Compton process of primary and scattered photons, is increasing. As result, the energy fluence is shifted towards lower energies (red histogram in Fig. 3(b) with a wide maximum at 1.2 MeV to 2.0 MeV).

The angular distributions of the electrons in vacuum are similar for short and large distances (Fig. 4). However, the tail registered at short distances is not present in the case of the extended distance and the distribution is confined below 20°. Large angle electrons at short distance may fall outside the scoring area at the large distance (i.e., geometrical effect). In the presence of air, the number of forward peaked electrons is decreasing and the contribution of the electrons scattered in larger solid angles is increasing. The tail is higher for the thicker air layer (SSD=445 cm).

Furthermore, variations of the mean energy of the electrons for different off‐axis positions are larger at the short distance compared to the extended distance (accelerator head surrounded by air). The mean energy of the electrons decreases from 3.5 MeV at the beam axis to 2.5 MeV at 17 cm radial distance as obtained from the analysis of the phase space at 90 cm from the accelerator target. Analogous variation for the phase space produced at 445 cm is from 2.6 MeV to 2.3 MeV at the beam axis and at 84 cm off‐axis, correspondingly. The dependence of the mean energy of the electrons on the off‐axis position is not shown here.

The presence of a spoiler changes considerably the shape of the electron fluence and of the angular distribution at the extended distance (Fig. 3(b), the green histograms). The head scatter electrons and electrons generated in the air are absorbed by the spoiler as discussed in Kassaee et al.^(5)^ Thus, the distributions in case of spoiler reflect the characteristics of the electrons produced in the spoiler. The mean energy of the electrons for different off‐axis positions at 445 cm behind a spoiler varies from 2.3 MeV to 2 MeV (i.e., it is lower than in absence of spoiler at the same distance (not shown here)). This is in line with the fact that high‐energy head scatter electrons may reach large distances in the air, but are absorbed by the spoiler.

**Figure 5:**
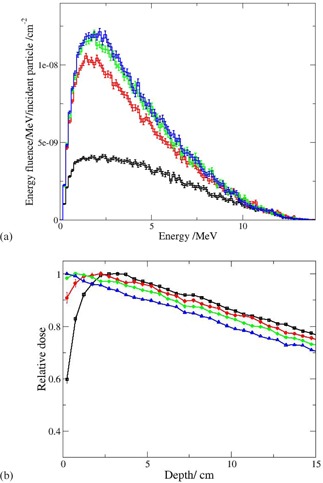
Energy fluence distribution (a) of electrons at 445 cm distance (black histogram: without spoiler; red histogram: 0.8 cm PMMA spoiler; green histogram: 1.6 cm PMMA spoiler; blue histogram: 2.4 cm PMMA spoiler); (b) depth dose distribution of electrons at 445 cm distance (black line: without spoiler; red line: 0.8 cm MMA spoiler; green line: 1.6 cm PMMA spoiler; blue line: 2.4 cm PMMA spoiler).

Electron energy fluence at 445 cm behind PMMA spoilers with different thicknesses is presented in Fig. 5(a). The energy fluence without spoiler, given for comparison, is much lower, which illustrates the role of the spoiler as an electron producer. When increasing the spoiler thickness from 0.8 cm to 1.6 cm, larger energy fluence is obtained. Further increase, however, does not have such an effect, due to the increased amount of self‐absorbed electrons. The effect of the spoiler thickness on the superficial dose is illustrated in Fig. 5(b). The depth distributions in the figure are normalized to dose maximum. The presence of 0.8 cm thick spoiler increases the superficial dose from 60% to 90%. The superficial dose for the 2.4 cm spoiler is 1.7% higher than that for the 1.6 cm spoiler as a result of the dominating energy fluence shown in Fig. 5(a). The attenuation is stronger for the 2.4 cm spoiler compared to that for the 1.6 cm spoiler. The dose uniformity for bilateral irradiation is slightly improved for the 1.6 cm spoiler. For example, the maximum/minimum dose ratio is 1.08 and 1.07 for 2.4 cm and 1.6 cm spoilers, respectively, assuming bilateral irradiation of a 40 cm thick water slab. When assuming a bilateral irradiation of a 30 cm thick water slab, the trend is similar. Typical reference width of an adult patient is 35 cm−40 cm. In general, the difference is small and both spoilers may be regarded as adequate. Nonetheless, a 1.6 cm thick spoiler is considered in our clinic as optimal. The depth distribution results are in agreement with the measurements in Gerig et al.,^(9)^ where the dose difference in the superficial region for 0.5 cm and 1.25 cm is found to be much larger compared to that for 1.25 cm and 2.4 cm spoiler. The analysis in the reference cited is for another beam energy (18 MV), SSD, and field size, thus the comparison is strictly qualitative.

**Figure 6:**
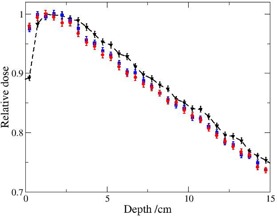
Depth dose distributions in water at SSD=480 cm behind 1.6 cm PMMA spoiler (red circles: STSD=5 cm; blue squares: STSD=10 cm, black triangles: STSD=30 cm).

**Figure 7:**
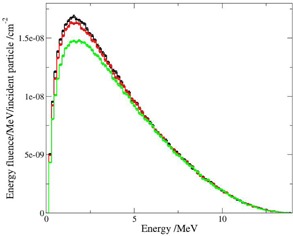
Energy fluence distribution for electrons at 480 cm distance behind 1.6 cm PMMA; black, red, and green lines for STSD=5, 10, and 30 cm, correspondingly.

Results for a PMMA spoiler of 1.6 cm thickness assuming different STSD are illustrated (Figs. 6 and 7). The effect of the spoiler on the dose in the buildup region is sufficient for STSD=5 cm and STSD=10 cm (Fig. 6) (i.e., the dose at 0.25 cm is nearly equal to the bulk dose at 3 cm). Even in the case of STSD=15 cm, the dose obtained at 0.2 cm was 99% compared to 3 cm depth, according to MC calculations and experimental results reported earlier.^(11)^ It is seen in Fig. 6, however, that for STSD=30 cm, the superficial dose at 0.25 cm is 10% less than the bulk dose. This behavior is comparable with the results for different air gaps reported in the study by Satory,^(16)^ where a 3 cm thick spoiler suspended above a water surface at SSD=100 cm is applied. Phase space analysis complements the depth dose observations. The energy fluence of the electrons for STSD=30 cm is lower compared to smaller distances (Fig. 7).

Typically, the distance between the spoiler and different parts of the body of the patient is not constant. Treatment protocols requiring a fixed distance to the patient central sagittal plane, as in our clinic, would result in various STSD distances for different patient sizes.

## IV. CONCLUSIONS

The role of a spoiler as a source of electrons at extended distances is investigated. Spoiler thickness of 1.6 cm is found to be optimal compared to thicknesses of 0.8 cm and 2.4 cm, assuming PMMA material. The dose at 0.25 cm depth in water is approximately 98%, compared to 3 cm depth for STSD up to 15 cm. However, it decreases to 90% for STSD=30 cm. Thus, the effect of the spoiler on the dose in buildup region is sufficient for STSD less or equal than 15 cm.

The STSD variations should be considered when treating patients, in particular when the treatment protocols are based on a fixed distance to the patient central sagittal plane, and also in order to maintain high dose in the superficial region. Therefore, spoiler holders at various distances to the patient central sagittal plane should be available. Special care should be taken for children.

The analysis of the energy fluence and angular distribution of the electron component of the beam may contribute to a better understanding of the beam characteristics at large distances prior to an eventual transition from a traditional bilateral beam delivery to a more sophisticated irradiation technique at isocenter distances.
